# A new chitinase-like xylanase inhibitor protein (XIP) from coffee (*Coffea arabica*) affects Soybean Asian rust (*Phakopsora pachyrhizi*) spore germination

**DOI:** 10.1186/1472-6750-11-14

**Published:** 2011-02-07

**Authors:** Erico AR Vasconcelos, Celso G Santana, Claudia V Godoy, Claudine DS Seixas, Marilia S Silva, Leonora RS Moreira, Osmundo B Oliveira-Neto, Daniel Price, Elaine Fitches, Edivaldo XF Filho, Angela Mehta, John A Gatehouse, Maria F Grossi-De-Sa

**Affiliations:** 1 Embrapa Recursos Genéticos e Biotecnologia. Parque Estação Biológica - PqEB - Av. W5 Norte (final). Postal box 02372 - Brasília, DF- 70770-917 - Brasil; 2 Embrapa Soja. Rod. Carlos João Strass - Distrito de Warta. Postal box 231 - Londrina, PR- 86001-970 - Brasil; 3 Embrapa Cerrados. BR 020 Km 18. Postal box: 08223 - Planaltina, DF- 73310-970 - Brasil; 4 Laboratório de Enzimologia, Departamento de Biologia Celular, Universidade de Brasília (UnB). Campus Universitário Darcy Ribeiro, DF - 70910-900, Brasília; 5 School of Biological and Biomedical Sciences, Durham University, South Road, Durham, DH1 3LE, UK

## Abstract

**Background:**

Asian rust (*Phakopsora pachyrhizi*) is a common disease in Brazilian soybean fields and it is difficult to control. To identify a biochemical candidate with potential to combat this disease, a new chitinase-like xylanase inhibitor protein (XIP) from coffee (*Coffea arabica*) (CaclXIP) leaves was cloned into the pGAPZα-B vector for expression in *Pichia pastoris*.

**Results:**

A cDNA encoding a chitinase-like xylanase inhibitor protein (XIP) from coffee (*Coffea arabica*) (CaclXIP), was isolated from leaves. The amino acid sequence predicts a (β/α)_8 _topology common to Class III Chitinases (glycoside hydrolase family 18 proteins; GH18), and shares similarity with other GH18 members, although it lacks the glutamic acid residue essential for catalysis, which is replaced by glutamine. CaclXIP was expressed as a recombinant protein in *Pichia pastoris*. Enzymatic assay showed that purified recombinant CaclXIP had only residual chitinolytic activity. However, it inhibited xylanases from *Acrophialophora nainiana *by approx. 60% when present at 12:1 (w/w) enzyme:inhibitor ratio. Additionally, CaclXIP at 1.5 μg/μL inhibited the germination of spores of *Phakopsora pachyrhizi *by 45%.

**Conclusions:**

Our data suggests that CaclXIP belongs to a class of naturally inactive chitinases that have evolved to act in plant cell defence as xylanase inhibitors. Its role on inhibiting germination of fungal spores makes it an eligible candidate gene for the control of Asian rust.

## Background

The plant surface is a complex molecular battlefield during plant-pathogen or plant-pest interaction. During infection, plant cells produce a group of proteins, coded by non-homologous genes, named Pathogenesis Related (PR) Proteins. Seventeen PR-proteins families have been identified based on biological activity, which can range from cell-wall/membrane degrading enzymes, to protease inhibitors, and proteins related to oxidative metabolism [1]. Each PR-protein family has a specific role during plant-pathogen interaction. Some of them act as "attack" molecules to damage the pathogen, while others act as "defence" molecules, to protect plant cells from the molecular attack of pathogens. Villamil and Hoorn [2] review aspects of this "zig-zag" model of plant-pathogen interaction.

Xylanase inhibitor proteins (XIP) are potential "defence" molecules, which could act to prevent plant cell wall degradation by fungal hydrolytic enzymes. They have sequence similarity to glycoside hydrolases of family 18 (GH18) that are plant class III chitinases (PR-8). The GH18 family includes naturally inactive chitinases showing (β/α)_8 _topology that are predicted to show no catalytic activity due to mutations in the catalytic domain. Some of these proteins have been identified as inhibitors of xylanases (belonging to glycoside hydrolase families GH10 and GH11). In wheat, a chitinase-like xylanase inhibitor protein (XIP-I) had its structure elucidated and its mechanism of inhibition proposed [3,4]. Structural features of these (β/α)_8 _chitinase-like xylanase inhibitors, as well its interaction with xylanases, has been reviewed recently [2].

Asian rust (*Phakopsora pachyrhizi*) is a new devastating disease, which has affected the cultivation of soybean (*Glycine max *(L.) Merril L) in Brazil. It was first detected in the country by 2001 and, due to favourable climatic conditions for fungal transmission, the productivity of the soybean crop, in yield/ha, declined by 17% from 2003 to 2005 [5,6]. Since the appearance of Soybean rust in Brazil, chemical fungicides from the group of Triazoles, Strobilurins and Benzimidazoles have been used for the control of this disease. However, the use of these fungicides is related to neurological, immunological and reproductive disorders in mammals, as well as causing arrest of mitosis [7,8]. Alternative, less environmentally-damaging methods for control of this pathogen that do not pose risks to human health are urgently required.

In this paper we report cloning, heterologous expression and enzymatic features of a new chitinase-like xylanase inhibitor protein (XIP) from coffee (*Coffea arabica*) (CaclXIP - *Coffea arabica *Chitinase-like Xylanase Inhibitor Protein), originally identified in the coffee genome [9] as a Class III Chitinase. CaclXIP showed only residual chitinolytic activity, but was an effective inhibitor of *Acrophialophora nainiana *xylanases, which are important enzymes to phytopathogenic fungi virulence. When assayed towards *P. pachyrhizi *(Asian rust), CaclXIP was able to arrest spore germination. As far as we know, this is the first time that a XIP-like molecule has been related to such biological activity. This work suggests that CaclXIP may be an eligible candidate for biotechnological approaches to control Asian rust. Such work is also trying to shed new light on the functional versatility of GH18 members and, consequently, the implication of such plurifunctionality for genome annotations and prediction of gene function.

## Results and Discussion

### Cloning, heterologous expression and purification of CaclXIP

**Figure 1:**
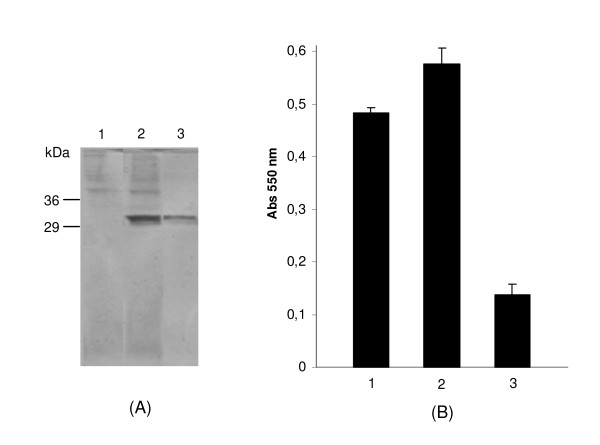
**Evaluation of chitinolytic activity of a new protein from *Coffea arabica *(CaclXIP) after heterologous expression**. (A) SDS-PAGE (12.5% acrylamide gel) of the different fractions obtained from CaclXIP expression. 1. Proteins from the culture supernatant inoculated with *P. pastoris *bearing an empty vector; 2. Proteins from the culture supernatant inoculated with *P. pastoris *bearing pGAPZα-B/*caclxip*; 3. CaclXIP eluted from ion exchange chromatography with 350 mM NaCl. Proteins were precipitated from 200 μL of each fraction with 4 volumes of cold acetone, solubilized in 50 μL of sample buffer and 10 μL were load on gel. A protein with molecular weight around 32 kDa was purified by ion exchange chromatography. The gel was stained with Coomassie Blue. (B) Chitinolytic assay. Each sample from (A) was dialyzed against reaction buffer (80 mM sodium phosphate buffer pH 6.8) and five micrograms of each sample were used per assay. Numbers 1, 2 and 3 are as described in (A). The same assay was performed at pH 4.8, 5.8, 7.8, and 8.8, without any significant variation.

Analysis of sequences present in the Coffee Genome Data Bank identified a type III chitinase-like gene, present in contig 14550, which codes to a xylanase inhibitor protein. A cDNA corresponding to this gene, designated *caclxip*, was cloned by RT-PCR techniques from RNA prepared from coffee leaves. The amino acid sequence predicted from the fragment cloned encodes a 32 kDa protein (pI 5.5) which differs from the predicted sequence present in contig 14550 by four amino acid substitutions (Arg125Ser, Met231Ile, Gly264Arg, Gly276Asp) and an insertion of Thr-Ile downstream of Ser279. This difference can be explained by natural genetic variation between coffee plant used in the preparation of cDNA library of Coffee Genome, and the one used in cloning procedures. However, according to modelling prediction, such substitutions do not disturb the (β/α)_8 _topology of GH18 members. The sequence coding for the mature protein, without plant signal peptide, was subcloned into a yeast expression vector (pGAPZα-B) arranged in frame with an N-terminal secretory signal (the yeast α-factor), and a C-terminal extension including a (His)6 tag. Recombinant protein was produced by heterologous expression in *Pichia pastoris*. After yeast transformation, a small scale expression assay was performed. One colony expressing a 32 kDa protein was selected for growth in a fermenter. After fermentation and recovery of culture supernatant, the heterologous protein was more efficiently purified from the 3 litres of culture supernatant by ion-exchange chromatography instead of metal chelate affinity chromatography. Approximately 70 mg of the recombinant CaclXIP protein was recovered, representating an expression level of 23 mg/l, a value more than four-fold higher than the one reported by Fitches and colleagues for production of an insect chitinase in *P. pastoris *[10]. Figure 1A shows analysis of the fractions from CaclXIP purification by SDS-PAGE gel electrophoresis.

### Comparison of CaclXIP with others GH18 and GH18-like proteins

**Figure 2:**
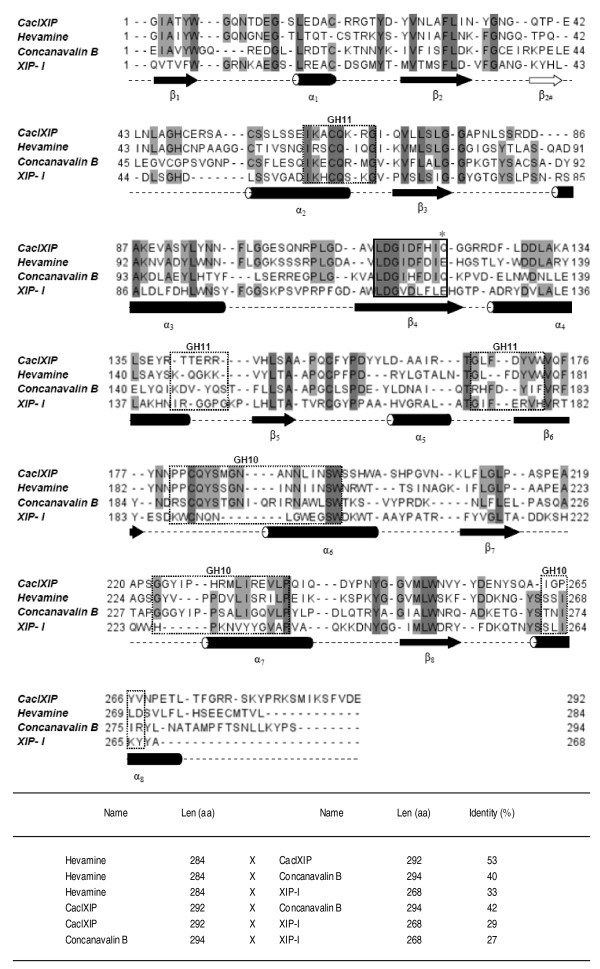
**Amino acid sequence alignment of selected GH18 plant members and secondary structure prediction**. Conserved residues are marked with a shadow. Region encompassing the catalytic domain of class III chitinases are marked with a continuous box. In CaclXIP the catalytic residue of GH18 (marked with *), a Glutamic acid (E), is replaced by a Glutamine (Q), much as in Concanavalin B. On the other hand, in XIP-I the glutamic acid in the active site is conserved, even though it does not show any chitinolytic activity. Sequences in the doted box show residues involved in complexing with GH10 or GH11 xylanases in XIP-I and its counterparts in the other proteins. Secondary structure alignment was performed with Jalview software and improved by hand. The table of sequence identity was obtained with free on line software ClustalW2 (http://www.ebi.ac.uk/Tools/clustalw2). Sequences of Hevamine [GenBank:DQ873889], Concanavalin B [Swiss-Prot:P49347], and XIP-I [Swiss-Prot:Q8L5C6] were obtained at NCBI (http://www.ncbi.nlm.nih.gov) data bank.

Plant Class III Chitinases belong to family 8 of pathogenesis related proteins (PR-8) [1]. This class of glycoside hydrolase proteins is characterised by a (β/α)_8 _topology and an active site containing two aspartic acid residues (D) and one glutamic acid residue (E) (as catalytically active residue) generally separated by phenylalanine and isoleucine (DxDxE) (Figure 2). Hevamine is a model plant Class III Chitinase, for which a full structure had been obtained by X-ray crystallography [11] and a catalytic mechanism established [12]. In the coffee genome data bank there are at least 4 contigs containing complete sequences predicted to encode Class III Chitinase proteins (contigs 18766, 7978, 14163, and 20121) showing 54 up to 57% of sequence identity with hevamine, including the catalytic domain (data not shown). The protein predicted by contig 14550 contains a catalytic site sequence "DFHIQ", where a glutamine residue replaces the catalytic glutamic acid (Figure 2). Such a substitution was demonstrated to be related to a lack of chitinolytic activity [13,14]. These data suggest that the sequence found in contig 14550 belongs to a group of naturally inactive plant class III chitinases, exemplified by Concanavalin B [15], that are thought to have recently evolved from PR-8 family. Members of this group of proteins have been shown to retain a function in plant cell defence through action as xylanase inhibitors.

Figure 2 shows the alignment of secondary structures of CaclXIP with other GH18 members. The β_4 _and β_8 _sheets are important regions to substrate interaction and chitinolytic activity [13]. Such regions are highly conserved in others GH18 members, even in those without enzymatic activity, probably due to maintainance of the (β/α)_8 _topology which is fundamental to xylanase inhibition activity [2]. Regions encompassing the former chitinase catalytic moiety and the predicted sites of interaction with xylanases GH10 and GH11 active sites are highlighted in figure 2. According to the alignment, CaclXIP shares more sequence identity with hevamine (53%) than with wheat XIP-I (29%), suggesting that CaclXIP belongs to a XIP group that had evolved from the GH18 family more recently than the group encompassing wheat XIP-I. Unlike CaclXIP, XIP-I retains the catalytic glutamic acid residue in the "active site" (Figure 2); however the side chain of this residue seems to be fully engaged in salt bridges with two neighbouring arginine residues (Arg158 and Arg181) [16], preventing wheat XIP-I from acting as a chitinase [3]. CaclXIP and wheat XIP-1 seems to reveal two different evolutionary routes for xylanase inhibition to arise from the GH18 family. One of the supposed routes is that of retaining catalytic potential, "Glu kept", where an intermediate molecule with chitinolytic activity as well xylanase inhibitory property may have been formed. A second route shows a loss of catalytic potential, "Glu lost", where the xylanase inhibitory property could emerge in a group of naturally inactivated chitinases, such as Concanavalin B.

### Evaluation of enzymatic role of CaclXIP in plant defence

Three samples representing stages of expression and purification of CaclXIP were assayed for chitinolytic activity (Figure 1B). The *P. pastoris *culture supernatant contains chitinase activity, but this is not significantly increased in recombinant yeast expressing CaclXIP. Purified CaclXIP contains no significant chitinolytic activity at pH 6.8 (Figure 1B). Repetition of the assays in a pH range from 4.8 to 8.8 neither showed any enzymatic activity. A 10-fold increase in CaclXIP concentration in combination with a doubling of the incubation time still did not show any enzymatic activity. The same lack of chitinase activity was observed by McLaughlan and colleagues in XIP-I from wheat [17]. The absence of significant chitinolytic activity in CaclXIP suggested that it might act as a proteinaceous inhibitor of xylanase, and this was confirmed when CaclXIP was used in an inhibition assay against complex xylanase fractions from *Aspergillus flavus*, *Aspergillus niger *and *Acrophialophora nainiana*. No inhibition activity was observed towards *A. flavus *xylanases, however CaclXIP inhibited xylanases from *A. nainiana *and *A. niger *by 56% and 19.5%, respectively, when assayed in an enzymes:inhibitor ratio of 12:1 (w/w) (Table 1).

**Table 1:** Evaluation of the xylanasic inhibition activity of CaclXIP towards enzymes of *Aspergillus niger *and *Acrophialophora nainiana*

Treatment	Xylanase activity (Abs 540 nm)	Inhibition (%)
*A. niger *xylanases	0.365 ± 0.057	Not applicable
*A. niger *xylanases + CaclXIP	0.294 ± 0.010	19.5 ± 3.4
*A. nainiana *xylanases	0.211 ± 0.018	Not applicable
*A. nainiana *xylanases + CaclXIP	0.092 ± 0.009	57 ± 9

* Xylanases from A. *niger *and *A. nainiana *(60 μg) were incubated with CaclXIP (5 μg) (enzymes:inhibitor ratio 12:1 [w/w]) as described in Material and Methods section. Xylanase activity and percentage inhibition are expressed as means + S. D., n = 3.

Different groups have reported that wheat XIP-I has no activity towards bacterial xylanases [18,16,19]. The activity of CaclXIP towards these enzymes is unknown, but the CaclXIP gene is highly transcribed in coffee leaves infected by the bacterial pathogen *Xylella fastidiosa *(data from Coffee Genome cDNA Library). The details of the mechanism of action of fungi and bacterial xylanases which could lead to such inhibition specificity were reviewed by Sunna and Antranikian [20]. Wheat XIP-I transcription can be induced by pathogens as well as by abiotic stress, such as wounding and methyl jasmonate treatment [21], although XIP-like genes are also expressed in tissues during growth and development of healthy wheat plants [22], as well as in response to pathogens [23]. In rice, three different XIPs were also induced by phytohormones and wounding in different tissues [24]. These results demonstrate that the role(s) of plant xylanase inhibitor proteins in plant metabolism as a whole remain largely obscure.

### Arrest of *Phakopsora pachyrhizi *spore germination by CaclXIP

When assayed at 1.5 μg/μL, CaclXIP was able to inhibit the germination of spores of *P. pachyrhizi *by 45% (Table 2). Although there are no other studies showing fungi spore germination arrestment by a XIP-like protein, the concentration at which CaclXIP has shown activity, besides high, can be considered significant when compared with the concentration at which 50% of *F. moniliforme *spores are affected (ED_50_) by a known antimicrobial peptide, like tobacco osmotin (ED_50 _0.8 μg/μL) [25]. Interestingly, inhibition of xylan degradation could not be the crucial factor for the arrestment to spore germination due to the absence of xylan in the assay reaction. In common with other phytopathogenic fungi, *P. pachyrhizi *uses a diversity of glycosidase enzymes to grow and infect plant cells [26] including cellulases, xylanases and mannanases. As far as we know, this is the first time that a XIP is reported to affect fungi metabolism other than through the disruption of xylan degradation.

**Table 2:** Evaluation of the activity of the CaclXIP on the germination of *Phakopsora pachyrhizi *spores

Treatment	Average of spore germination (%)
*Control*	99 ± 1
*CaclXIP 1.5 μg/μL*	55 ± 5

* Average percentage of germinated *Phakopsora pachyrhizi *spores upon incubation with either 10 mM Tris-HCl pH 6.8 (Tris-HCl) or purified CaclXIP diluted in 10 mM Tris-HCl pH 6.8 to the final concentration of 1.5 μg/μL (CaclXIP). Average spores germination is expressed as means + S. D., n = 3.

## Conclusions

The action of CaclXIP on germination of *P. pachyrhizi *spores represents a first approach towards a biotechnological solution to pathogen losses in soybean cultivation. Expression of this protein in transgenic soybean could substantially increase resistance, with low environmental impact. However, CaclXIP may only be one of a range of defensive proteins of this type that could be exploited. The protein superfamily that includes glycoside hydrolase family 18 contains proteins of similar topology but varying functional roles. These proteins can act at different stages of the "zig-zag" model of attack and counterattack between plant and pathogen [2], and a combination of different proteins could act synergistically in defence against pathogen attack. Besides being expressed in combination with other defensive proteins, CaclXIP activity could be increased by techniques of *in vitro *molecular evolution [27] and used to develop a GM Soybean resistant to Asian rust.

## Methods

### Cloning of a chitinase-like XIP gene from Coffee plants

*Coffea arabica *plants were cultivated in a green house for approximately 6 months. Leaves were used for RNA extraction with Trizol reagent (Invitrogen) and the cDNA was synthesized using First-Strand cDNA Synthesis Kit (Invitrogen). The cDNA encoding the complete chitinase-like XIP (CaclXIP) gene was amplified using primers designed according to the sequence of contig 14550 obtained from the Coffee Genome (http://www.lge.ibi.unicamp.br/cafe/), annotated as a Class III Chitinase: *pFwd *5' ATGGCTCCCTGTTTTAGA 3'; *pRev *5' TTACTCATCCACAAAAGA 3'. PCR conditions were: 5 min at 95°C; 45 s at 95°C; 45 s at 60°C; 1 min at 72°C (40 cycles) and a final extension of 5 min at 72°C. A fragment around 970 bp was amplified, cloned into pGEM T-Easy vector (Promega), according to manufacturer instructions, and further sequenced for confirmation.

### Subcloning of the region coding for mature CaclXIP in pGAPZα-B to constitutive expression in *Pichia pastoris*

pGEM T-Easy bearing CaclXIP gene from *C. arabica *was used as template. The region encompassing the signal peptide was delimited by SignalP 3.0 on line free software (http://www.cbs.dtu.dk/services/SignalP/) and primers were designed to subclone the region coding to the mature protein: *CaclXIPfwd *5' TAAGAATT*CAA*GCTGGAATTGCCACCTAC 3'; *CaclXIPrev *5' TTAGTCGACCTCATCCACAAAAGACTTTATCATG 3. *Eco*R I and *Sal *I restriction sites were inserted in 5` and 3` ends of forward and reverse primers respectively, and are shown above underlined. A codon to glutamine was inserted in the end of *Eco*R I restriction site to keep it in the correct frame (showed in italics). Glutamine was chosen to avoid the potential disruption of the physico-chemical properties of the former first amino acid. PCR conditions were: 30 s at 98°C; 10 s at 98°C; 10 s at 60°C; 45 s at 72°C (20 cycles) and a final extension of 5 min at 72°C. As expected, a fragment around 890 bp was amplified, sequenced, and inserted into pGAPZα-B vector previously digested with *Eco*R I and *Sal *I, allowing the synthesis of the heterologous protein with a His-tag in the C-terminal and without the Myc epitope supplied by the vector.

### *Pichia pastoris *transformation and heterologous expression of CaclXIP gene

Around 10 μg of pGAPZα-B/*caclxip *were used to transform competent cells of *P. pastoris *SMD1168 protease deficient by heat-shock. Plasmid was linearised with *Bln *I restriction enzyme and transformation carried out according to the protocol of the Pichia EasyComp™ Transformation kit (Invitrogen). Transformed cells were spread on YPG (1% [w/v] Yeast extract, 2% [w/v] peptone, 4% [v/v] glycerol)/Zeocine 100 μg/mL medium, 1.5% Agar and kept at 28°C until emergence of colonies (2 to 4 days). Emerged colonies were used in a small scale expression assay, where each colony was used to inoculate 10 mL of YPG/Zeocine 100 μg/mL liquid medium. Cultures were kept at 28°C under 220 rpm agitation for 4 days. Supernatant of the cultures were checked by SDS-PAGE 12.5% [28] to detect the recombinant protein. One positive colony was selected to a large scale expression in a 3 litres BioFlo 110 laboratory fermenter (New Brunswick Scientific). Fermentation procedure occurs according to described by Fitches [29].

### Purification of recombinant CaclXIP

Supernatant from the fermentation was filtered through 0.2 μm and diluted 1:1 (v/v) in sodium acetate pH 4.0 to a final concentration of 50 mM. Recombinant proteins were purified by Ion Exchange chromatography in a 25 mL S-Sepharose (G.E. Healthcare) column previously equilibrated with 50 mM Sodium acetate pH 4.0 at 2 mL/min. Binding proteins were eluted with a salt gradient of 0 - 1 M NaCl. Recombinant protein was eluted at approximately 350 mM NaCl and checked for purity by SDS-PAGE 12.5% [28]. Five millilitres of the combined column fractions containing recombinant proteins were dialysed against 80 mM sodium phosphate buffer pH 6.8 and tested for chitinolytic activity. The rest of the material was dialysed against ammonium bicarbonate and freeze dried. The concentration of the purified protein was estimated by comparison with known amounts of a standard protein by SDS-PAGE, as described by Fitches [29].

### Chitinolytic activity assay

To detect chitinolytic activity, three samples were dialyzed in 80 mM sodium phosphate buffer pH 6.8. Samples were: supernatant of a culture inoculated with pGAPZα-B empty, supernatant of a culture inoculated with pGAPZα-B/*caclxip*, and CaclXIP purified. To 300 μL of all samples, containing 5 μg of total protein, were added 200 μL of the substrate CM-chitin-RBV (Hornik) (1 mg/mL) and assay was performed as described by Fitches [10]. The assay was repeated with incubation time 2-fold higher and CaclXIP concentration 10-fold higher as well as at pH 4.8, 5.8, 7.8, 8.8.

### Xylanase inhibition assay

Protein fraction with xylanase activity was obtained from, *Aspergillus flavus*, *Aspergillus niger *and *Acrophialophora nainiana *according to Salles [30]. Inhibition reaction was performed in a final volume of 150 μL containing 1% (w/v) commercial oat spelt xylan, 60 μg xylanases, and 5 μg CaclXIP. Reactions were kept at 50°C for 30 min and than 300 μL of DNS reagent [31] was added following boiling for 10 min. Subsequently, 1.5 mL of distillated water was added and absorbance was measured at 540 nm. Assays were repeated in triplicate.

### Bioassay towards *Phakopsora pachyrhizi *spores

Soybean plants infected by *P. Pachyrhizi *were maintained in greenhouse conditions to be used as source of spores. For that purpose, infected leaves were gently tapped and naturally detached spores were collected underneath the leaves onto a white paper sheet. The collected spores were immediately diluted into sterile distilled water containing 0.01% (v/v) Tween 20 to the concentration of 2 × 10^4 ^spores/mL. Purified CaclXIP was diluted in 10 mM Tris-HCl pH 6.8 to the concentration of 3.0 μg/μL. The bioassays were set up in microplates of 96 wells, with three replicates per treatment. The treatment to test inhibition of spore germination by CaclXIP consisted of 50 μL of CaclXIP 3.0 μg/μL mixed to 50 μL of 2 × 10^4 ^spores/mL. The negative control treatment consisted of 10 mM Tris-HCl pH 6.8 replacing CaclXIP. The microplates were then incubated at 25°C in a humid chamber overnight. To terminate the assay, lactofenol was added to the wells and germinated spores were counted upon observation at a stereomicroscope within several fields of 100 spores each field.

### Homology modelling of CaclXIP and comparison with others GH18 members

To the prediction of CaclXIP secondary structures, PDB files were generated using 3D-JIGSAW on line software (http://bmm.cancerresearchuk.org/~3djigsaw/). Alignment between the mature region of CaclXIP, Hevamine [GenBank:DQ873889], Concanavalin B [Swiss-Prot:P49347], and XIP-I [Swiss-Prot:Q8L5C6)], was performed using the structural alignment tool of Jalview 2.4 software [32] and improved by hand until a satisfactory placement of conserved blocks and amino acid identities was obtained. The model was validated with PROCHECK [33].

## Authors' contributions

CGS and AM: Carried out the cloning of CaclXIP from coffee leaves. CVD, CDSS and MSS: Carried out the bioassay of spore germination inhibition. LRSM and EXFF: Carried out the preparation of xylanasic fractions from *A. niger*, *A. flavus *and *A. nainiana *for inhibition assay. EARV and LRSM: Carried out the enzymatic inhibition assays. EARV, OBON and DP: Participate in the procedures to subclone mature CaclXIP in a *Pichia *expression vector. EARV and EF: Carried out the purification of recombinant CaclXIP and assays to detect chitinolitc activity. EARV: Carried out the homology modeling and secondary structure prediction of CaclXIP. MFGS and JAG: Advisors and group leaders.

All authors read and approved the final version of this manuscript.
